# Participation in a 20‐Year Randomized Dietary Trial and University Enrollment

**DOI:** 10.1002/hsr2.72359

**Published:** 2026-04-19

**Authors:** Jutta Viinikainen, Guido Heineck, Petri Böckerman, Katja Pahkala, Antti Jula, Jaana T. Kari, Hanna Lagström, Harri Niinikoski, Jaakko Pehkonen, Suvi P. Rovio, Tapani Rönnemaa, Pia Salo, Jorma Viikari, Olli Raitakari

**Affiliations:** ^1^ Jyväskylä University School of Business and Economics, University of Jyväskylä Jyväskylä Finland; ^2^ Department of Economics University of Bamberg Bamberg Germany; ^3^ IZA Network at LISER IZA Institute of Labor Economics Luxembourg Germany; ^4^ Labour Institute for Economic Research LABORE Helsinki Finland; ^5^ Centre for Population Health Research University of Turku and Turku University Hospital Turku Finland; ^6^ Research Centre of Applied and Preventive Cardiovascular Medicine University of Turku Turku Finland; ^7^ Paavo Nurmi Centre and Unit for Health and Physical Activity University of Turku Turku Finland; ^8^ Finnish Institute for Health and Welfare Helsinki Finland; ^9^ Faculty of Medicine University of Turku Turku Finland; ^10^ Department of Public Health University of Turku Turku Finland; ^11^ Nutrition and Food Research Center, Faculty of Medicine University of Turku Finland; ^12^ Institute of Biomedicine, Integrative Physiology and Pharmacology University of Turku Turku Finland; ^13^ Department of Paediatrics and Adolescent Medicine University of Turku Turku Finland; ^14^ Division of Medicine Turku University Hospital Turku Finland; ^15^ Department of Clinical Physiology and Nuclear Medicine Turku University Hospital Turku Finland

**Keywords:** diet, education, family background, randomized controlled trial, university enrollment

## Abstract

**Background and Aims:**

A nutritionally balanced, age‐appropriate diet is crucial for child development. The randomized Special Turku Coronary Risk Factor Intervention Project (STRIP) was a 20‐year dietary intervention starting at 7 months of age. It aimed at improving fat quality and promoting healthy foods to prevent cardiovascular diseases. We examined whether the intervention was associated with university enrollment and difficulties in school‐related tasks in a *post hoc* setting.

**Methods:**

Participants (*n* = 1062) were recruited at the age of 5 months between December 1, 1989, and May 30, 1992, from child health clinics in Turku, Finland, and were randomly assigned to either the intervention (*n* = 541) or control group (*n* = 521) at the age of 7 months. Children in the intervention group received personalized dietary counseling through age 20, without a fixed diet. We used linear probability models estimated by ordinary least squares (OLS) to investigate whether being in the intervention group was associated with university enrollment by age 26, and OLS regressions to examine its association with difficulties in school‐related tasks at age 10. The study is reported in accordance with the CONSORT guidelines.

**Results:**

In total, 639 participants provided university enrollment data. Among males from low‐education families, being in the intervention group was associated with a 32 percentage point higher likelihood of enrollment (*b* = 0.324, *p* = 0.004, 95% confidence interval (CI): 0.103, 0.546). Among all males the association was 11 percentage points (*b* = 0.107, *p* = 0.07, 95% CI: −0.007, 0.220), and among females −3 percentage points (*b* = −0.033, *p* = 0.53, 95% CI: −0.137, 0.071). The results also suggested that diet, rather than cardiovascular health, may serve as a mediator.

**Conclusions:**

Long‐term dietary counseling is associated with increased educational attainment among males from low‐education families. The study is registered at ClinicalTrials.gov (#NCT00223600).

## 1 Introduction

An age‐appropriate and nutritionally balanced diet is crucial for child growth and development. Nutrient deficiencies are associated with poorer health [1], and improvements in nutrition can enhance child health even in high‐income countries [2]. Nutrition has also been linked to academic performance. Prior studies suggest that better‐nourished children tend to perform better in school in low‐income countries [3], and children in high‐income countries may likewise benefit from improved nutrition. Eating breakfast has been associated with better cognitive and academic performance among children and adolescents [4, 5, 6], and the provision of school breakfasts [7, 8] or lunches [9, 10] has been shown to improve academic test results. Moreover, improvements in school meal quality [11] and an increase in their energy content [12] have been linked to better scores on academic tests. Nutritional risks in early childhood have additionally been connected to a higher likelihood of schools reporting concerns about a child's development or behavior [13]. Although many studies have linked the provision of school meals to better short‐term educational outcomes, a recent meta‐analysis suggests that these effects are, on average, relatively small. The findings also indicate heterogeneity in program outcomes, with positive impacts more likely for breakfast initiatives and means‐tested schemes. In addition, some evidence points to sex differences in responses, with female students showing less favourable outcomes than males [14].

While evidence on the short‐term academic benefits of school meal programs is extensive, much less is known about the medium‐ and long‐term impacts of dietary improvements in high‐income countries [14]. Yet previous research suggests that these longer‐term effects can be substantial. For example, the introduction of free, nutritious school lunches in Sweden in the 1950s and 1960s improved the intake of proteins, minerals, and vitamins, contributing to better health and cognitive development. Consistent with this, pupils who were exposed to the school lunch program had better health, higher educational attainment, and greater earnings later in life [15].

We contribute to the literature by examining whether a long‐term dietary intervention, the Special Turku Coronary Risk Factor Intervention Project (STRIP), was associated with subsequent educational performance. The intervention is unique in that it began in infancy and continued for 20 years, with personalized dietary counseling provided to children and their parents in the intervention group. Our analysis represents a *post hoc* extension of a randomized controlled trial originally designed to study cardiovascular outcomes, and the educational outcomes examined here were not primary endpoints of the intervention. Attrition over the long follow‐up period reduced the estimation sample, and some analyses focus on relatively small subgroups. Accordingly, the estimates are most appropriately interpreted as associations leveraging randomized assignment. However, our study makes four key contributions. First, we investigated whether the intervention had a long‐term association with educational outcomes by examining whether being in the intervention group was associated with university enrollment by age 26. Second, we examined whether being in the intervention group was associated with the level of difficulty the child experienced with school‐related tasks at age 10. Third, we explored potential differences in the results by sex and parental socioeconomic status (SES). Fourth, we examined the role of improved diet and cardiovascular risk profile as potential mediators between the intervention and university enrollment.

## 2 Methods

### 2.1 Study Design and Participants

The STRIP study was a prospective randomized controlled trial (RCT) conducted in Turku, Finland, starting in 1990. The study was motivated by the high incidence of coronary artery disease among Finnish adults and elevated cholesterol levels observed in boys. High serum and LDL cholesterol levels in childhood are linked to adverse cardiovascular changes later in life. These risks can be reduced through healthy living habits, which may be easier to adopt in early childhood than in adulthood [16]. The STRIP project aimed to reduce cardiovascular disease risk factors by providing repeated dietary and other lifestyle counseling to intervention group children and their families. The main aim was to improve the quality of fats in children's diets. The secondary aim was to promote the intake of fruit, vegetables, and whole‐grain products [17]. The intervention led to lower intakes of saturated fats, a more favourable ratio of unsaturated to saturated fatty acids [17], and better diet quality in terms of fibre, fruit, and vegetable intake [18]. Furthermore, compared with controls, the intervention resulted in higher intakes of several key vitamins and minerals [18].

**Figure 1:**
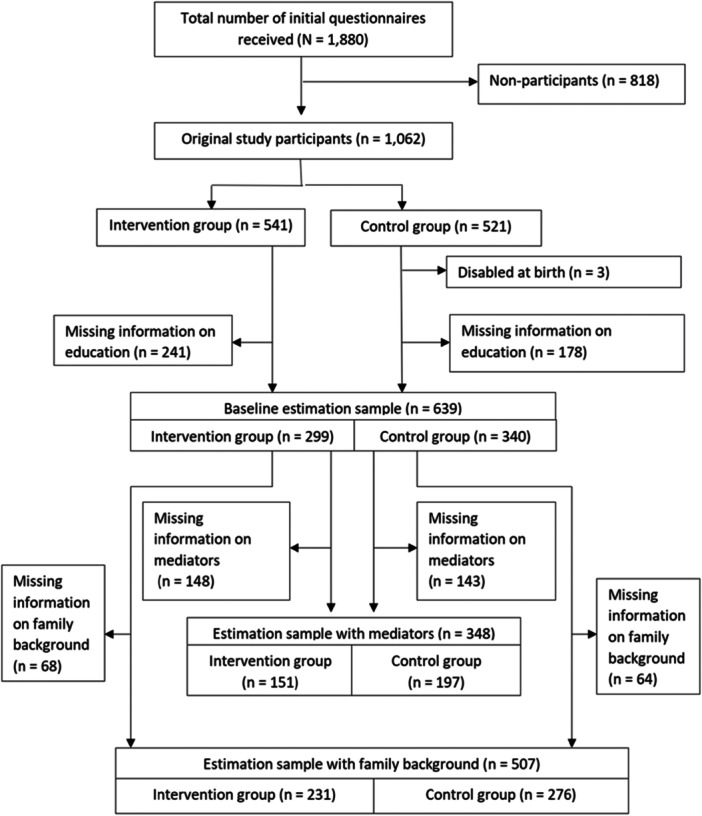
Flow chart of the Study.

The STRIP study enrolled children and their families who were recruited at well‐baby clinics at their routine 5‐month visit between December 1, 1989, and May 30, 1992 in the city of Turku, Finland. The children constituted a representative sample of 5‐month‐old children in the area. A total of 1062 7‐month‐old infants and their parents were enrolled in the trial. At 7 months of age, the infants were randomly assigned to either the intervention group (*n* = 541) or the control group (*n* = 521). Participants assigned to the intervention group received regular dietary counseling. It started after the randomization and continued until the participants were 20 years old. Initially, the counseling was given to the parents, but from the age of 7 it was aimed directly at the children. Due to attrition and missing information, the estimation sample was smaller than the total sample. Figure 1 shows the flow chart of study subjects.

This study involved human participants. Written informed consent was obtained from the parents at the beginning of the study and from the children at ages 15 and 18. The study followed the Declaration of Helsinki, was approved by the Joint Commission on Ethics of Turku University and Turku University Central Hospital, and was registered with ClinicalTrials.gov (NCT00223600). The study was reported in accordance with the Consolidated Standards of Reporting Trials (CONSORT) guidelines. ChatGPT was used for language editing.

### 2.2 Procedures

Both groups met regularly with a nutritionist and a paediatrician or a nurse during the intervention. Children in the intervention group visited the study centre every 1 to 3 months until the age of 2, and every 6 months thereafter. In comparison, children in the control group visited the centre twice a year initially, and then annually from the age of 7. While both groups met the same health staff, only the intervention group received dietary counseling. All children received standard health care education at well‐baby clinics initially and later at school [17].

Children in the intervention group received personalized dietary counseling but were not prescribed a specific diet. The aim was to enhance parents' and children's nutritional knowledge, with a particular focus on improving fat quality by replacing saturated fats with unsaturated fats. Moreover, target values for energy intake from proteins, carbohydrates, and fats were used as guidelines for diet quality. Increasing the consumption of whole‐grain products, fruits, and vegetables as well as reducing sodium and sucrose intake, was also recommended. The dietary advice was based on the prevailing Nordic nutrition recommendations. Although these recommendations have been updated over time, changes regarding total fat intake and fat composition, which were the main targets of the STRIP intervention, have been relatively modest, and the underlying principles have remained largely stable (see Supporting Information S1: Table S1). In particular, the emphasis on limiting saturated fat and ensuring an adequate intake of polyunsaturated fatty acids (omega‐6 and omega‐3) has been consistent across guideline versions. Information from food records was submitted by the participants before their study visit. Food records were also maintained for children in the control group, but they were not discussed during meetings [18].

### 2.3 Measures

#### 2.3.1 Outcome and Explanatory Variables

The main outcome variable was self‐reported university enrollment by age 26. It was coded as 1 if the participant was enrolled in or had completed a university‐level education (university or university of applied sciences) by age 26, and 0 otherwise. For university enrollment, data at age 20 were used if information at age 26 was not available (*n* = 245). We also examined the level of difficulty the child experienced with school‐related tasks at age 10. The indicator was a summary score calculated by summing four self‐assessed items, each rated on a 3‐point scale (0 = no; 1 = some; 2 = major) reflecting difficulties in following teaching, completing homework assignments, reading tasks, and writing tasks.

The main independent variable of interest was an indicator equal to 1 for individuals in the intervention group and 0 otherwise. As an additional control variable, we included an indicator for sex assigned at birth, which was coded as 1 for females and 0 for males.

We also classified families by parental SES to allow for subgroup analyses. SES was determined based on parents' reports of their educational attainment when the child was 5 years old. Families in which at least one parent had completed university‐level education were categorized as high SES, and those in which neither parent had done so were categorized as low SES.

#### 2.3.2 Mediators

Because the primary objective of the intervention was to improve children's diets and reduce cardiovascular risk factors, we used two measures of diet quality (diet score and quality of fat intake) and two measures related to cardiovascular health (ideal cardiovascular health index and a variable indicating metabolic syndrome [MetS]) as potential mediators of the link between the intervention and university enrollment.

The diet score was calculated based on food records collected annually from age 13 months, initially completed by parents and later by the children themselves. At each study visit, the nutritionist checked the records for completeness and accuracy. Food intake was analyzed using Micro‐Nutrica software and categorized into seven favourable (fiber‐rich grain products, fruits and berries, vegetables, fish, nuts and seeds, low‐fat unsweetened dairy, vegetable‐oil based fats) and four unfavourable (red and processed meat, sugar‐sweetened beverages, salty snacks, desserts) food groups. Participants were assigned points based on their consumption levels, with higher scores reflecting healthier diets. The total diet score ranged from 0 to 33, with higher values indicating better dietary quality [18]. In this study, we used an average of diet score values between ages 13 months and 17 years as the indicator of long‐term diet quality.

Fat quality was assessed relative to the RCT's target ratio of 1:2 for saturated to unsaturated fatty acids, evaluated annually from 13 months onward using food records. A binary variable indicated whether the target was met, and a long‐term measure was calculated as the average achievement of this target from 13 months to 17 years.

An ideal cardiovascular health index was created based on seven metrics: smoking, body mass index, physical activity, diet, total cholesterol, glucose, and blood pressure, following American Heart Association guidelines [19]. Smoking habits, leisure‐time physical activity, and dietary intake were self‐reported by adolescents via questionnaires and food records, while BMI was calculated from measured height and weight, cholesterol and glucose were measured from fasting blood samples, and blood pressure from two seated readings using an oscillometric device. Each metric was scored as 1 if the ideal criterion was met and 0 otherwise, resulting in a total score from 0 to 7, with higher values indicating better cardiovascular health [20]. We used the average index value from ages 15 to 17, as data were available starting at age 15.

The MetS indicator was based on five characteristics: waist circumference, blood pressure, glucose, triglycerides, and HDL cholesterol. Waist circumference was measured at the midline between the lowest rib and iliac crest, and blood pressure was measured twice in a seated position after at least 15 min of rest, with the mean used for analysis. Weight and height were measured to calculate BMI. Fasting venous blood samples were collected to determine plasma glucose, triglycerides, and HDL cholesterol using standard enzymatic methods. A participant was classified as having MetS if three or more components exceeded recommended levels, with the indicator scored as one in such cases and 0 otherwise [21]. We used the average MetS value from ages 15 to 17, as data on metabolic syndrome were available starting at age 15. In the estimation sample (*n* = 348), 25% of participants (20% in the intervention group and 28% in the control group) were classified as having MetS at least once during the observation period.

### 2.4 Statistical Analyses

We used a linear probability model (Ordinary Least Squares, OLS) to regress university enrollment (*E*) on the random intervention assignment (*I*) for individual *i* (Equation 1):

(1) $$E_{i}=\beta_{0}+\beta_{1}I_{i}+\varepsilon_{i}$$

We estimated linear probability models, as they facilitate interpretation and are less sensitive to distributional assumptions. For robustness, we also provided the main results based on logit model. To evaluate potential differences by sex, we estimated interaction models including all participants (Equation 2), as well as stratified models for low‐ and high‐SES groups. The variable *Female* was coded as 1 if the individual was female and 0 if male as described in the *Measures* section. In Equation 2, $\beta_{1}$ captures the association between being in the intervention group and university enrollment among males. For females, the association is the sum of $\beta_{1}$ and $\beta_{3}$.

(2) $$E_{i}=\beta_{0}+\beta_{1}I_{i}+\beta_{2}{\mathrm{Female}}_{i}+\beta_{3}(I_{i}\times {\mathrm{Female}}_{i})+\varepsilon_{i}$$

We applied the same OLS specifications to an alternative outcome measuring difficulties with school tasks at age 10. These models were also stratified by SES. The sex–SES interaction analyses were not planned *a priori* and are thus exploratory and based on a relatively small sample size. However, as nutrition‐related interventions may have particularly strong effects among disadvantaged groups, exploring these differences likely provides insight into potential heterogeneity across subgroups.

To investigate whether the relationship between the intervention and university enrollment was mediated by nutrition (i.e., diet score and fat intake quality) or by cardiovascular health (i.e., ideal cardiovascular health index and MetS) we augmented Equation 1 to include these potential mediators. This is an informal mediation approach based on changes in the intervention coefficient when adding potential mediators, rather than a full causal mediation analysis. The models were estimated for males and for low‐SES males. This focus reflects that no statistically significant association between the intervention and university enrollment was observed among females, while among males the intervention was associated with educational outcomes primarily in the low‐SES subgroup. To evaluate whether the estimated effect of the intervention changed after including the mediators, we tested the equality of the intervention coefficients between the basic and augmented models. This was done by computing point estimates for linear combinations of coefficients using the *lincom* command in Stata.

All analyses were performed using Stata version 19.5, with statistical significance defined as *p* < 0.05. All hypothesis tests were two‐sided. Heteroskedasticity‐robust standard errors were used in Tables 2 and 3, whereas Table 4 reports non‐robust standard errors because the *suest* command of Stata software package required for *lincom* does not support robust standard errors. Clustering was not necessary because the treatment is randomly assigned at the individual level and each participant contributes a single observation to the main outcome regressions, so there is no within‐cluster correlation that would require clustered inference.

## 3 Results

Table 1 presented the summary statistics. Of the original sample of participants (*n* = 1062), information on university enrollment was available for 639 participants, who constituted the estimation sample. The share of females was 54% both the intervention and control groups (intervention group: 162/299; control group: 184/340). Comparison of Panels A and B shows that the attrition in sample size was higher in the intervention group and among males. The proportion of participants with high SES was 71% (164/231) in the intervention group and 69% (190/276) in the control group (Panel C). Previous studies have shown that participants in the intervention group had, on average, healthier diets, better fat intake quality, lower prevalence of metabolic syndrome, and better ideal cardiovascular health indexes [16, 17, 19, 20]. Our descriptive statistics were in line with these results (Panel D).

**Table 1:** Summary statistics.

	(1)	(2)
	Intervention	Control
*Total sample*		
Panel A: Baseline characteristics		
Female	47.32%	49.14%
*N*	541	521
*Estimation sample*		
Panel B: Baseline characteristics		
Female	54.18%	54.12%
*N*	299	340
Panel C: Family background		
High family SES	71.00%	68.84%
*N*	231	276
Panel D: Observed diet‐ and health‐related outcomes		
Diet score (min 0, max 33)	17.25 (2.74)	14.95 (2.47)
Fat quality target achieved (0,1)	0.16 (0.07; 0.26)	0 (0; 0.05)
MetS (0,1)	0 (0; 0)	0 (0; 0.33)
Ideal cardiovascular health score (0–7)	4.5 (4; 5)	4.5 (3.5; 5)
*N*	151	197

*Note:* The table presents the shares of females and family background characteristics, the mean and standard deviation of the diet score variable, and the median with the 25th and 75th percentiles of other diet‐ and health‐related variables for the intervention (Column 1) and control (Column 2) groups.

Results from the pooled sample indicated no differences in university enrollment between the intervention and control groups (Table 2, Panel A). Among all males, the estimated association was 11% points (*b *= 0.107, *p* = 0.07, 95% confidence interval [CI]: −0.007, 0.220), whereas among females it was −3% points (*b* = −0.033, *p* = 0.53, 95% CI: −0.137, 0.071) (Panel B). Additional analyses (Panels C and D) suggested that participation in the intervention was associated with higher university enrollment, particularly among males from a low socioeconomic background. For these males, being in the intervention group was significantly associated with a 32 percentage point higher probability of enrolling in university compared with males in the control group (*b* = 0.324, *p* = 0.004, 95% CI: 0.103, 0.546). Results from a logit model were similar (Supporting Information S1: Table S2). Notably, participation in the intervention was also associated with fewer difficulties in school‐related tasks among low‐SES males (Table 3), which may partly explain the observed increase in university enrollment.

**Table 2:** Dietary intervention participation and university enrollment.

	Coefficient	*p*‐value	95% CI
Panel A: All (*n* = 639)			
Intervention	0.031	0.428	−0.046, 0.108
Panel B: All (*n* = 639)			
Intervention	0.107	0.066	−0.007, 0.220
Intervention × female	−0.140	0.075	−0.294, 0.014
Panel C: Low SES (*n* = 153)			
Intervention	0.324	0.004	0.103, 0.546
Intervention × female	−0.507	0.002	−0.818, ‐0.196
Panel D: High SES (*n* = 354)			
Intervention	0.033	0.676	−0.122, 0.188
Intervention × female	−0.084	0.427	−0.291, 0.124

*Note:* The table reports OLS coefficients, *p*‐values, and 95% confidence intervals from a regression of university enrollment on random intervention assignment (1 for individuals in the intervention group and 0 otherwise), using heteroskedasticity‐robust standard errors. The outcome variable equals one if the person has completed university‐level education or is enrolled in university by the age of 26. Panels B‐D include a sex indicator (male = 0, female = 1) and an interaction term (Intervention × female) as additional controls. Families with at least one parent who had completed university‐level education by the time the child was five years old are considered high SES families, while those without such a parent are considered low SES families.

Abbreviation: SES, socioeconomic status.

**Table 3:** Dietary intervention participation and difficulties with school tasks at age 10.

	Coefficient	*p*‐value	95% CI
Panel A: All (*n *= 583)			
Intervention	−0.178	0.084	−0.381, 0.024
Panel B: All (*n* = 583)			
Intervention	−0.276	0.062	−0.566, 0.014
Intervention × female	0.205	0.315	−0.195, 0.605
Panel C: Low SES (*n* = 146)			
Intervention	−0.713	0.025	−1.336, −0.090
Intervention × female	0.309	0.539	−0.682, 1.300
Panel D: High SES (*n* = 375)			
Intervention	−0.087	0.620	−0.430, 0.256
Intervention × female	0.227	0.326	−0.228, 0.683

*Note:* The table presents OLS coefficients, *p*‐values, and 95% confidence intervals from a regression of schooling difficulties on random intervention assignment (1 for individuals in the intervention group and 0 otherwise), using heteroskedasticity‐robust standard errors. The outcome variable is a summary measure, which indicates the level of difficulties with school‐related tasks at the age of 10. For additional notes, see Table 2.

The results presented in Table 4 examine diet and cardiovascular health as potential mediators of increased university enrollment. Given the results in Table 2, we focused only on males. Pairwise correlations among diet and cardiovascular mediators were small (≤ 0.12), indicating that potential multicollinearity is minimal, and models including the mediators separately or jointly yielded qualitatively similar results. The intervention point estimate lost its significance among low‐SES males when the diet indicators of a healthy diet but not those of the health were added to the explanatory model of university enrollment. These results suggested that diet, rather than cardiovascular health, may act as a potential mediator.

**Table 4:** Dietary intervention participation and university enrollment. Models augmented with potential mediators.

	Coefficient	*p*‐value	95% CI	Equality of intervention coefficients *p*‐values
Panel A: Male (*n* = 167)				
Basic model	0.119	0.126	−0.034, 0.273	—
Diet	0.101	0.309	−0.095, 0.297	0.761
Health	0.108	0.174	−0.048, 0.264	0.423
Diet and health	0.088	0.383	−0.111, 0.287	0.605
Panel B: Low SES male (*n* = 47)				
Basic model	0.363	0.014	0.078, 0.648	—
Diet	0.193	0.311	−0.187, 0.574	0.121
Health	0.344	0.022	0.052, 0.637	0.626
Diet and health	0.169	0.398	−0.231, 0.569	0.103

*Note:* The table reports OLS coefficients, *p*‐values, and 95% confidence intervals from a regression of university enrollment on random intervention assignment (1 for individuals in the intervention group and 0 otherwise), using conventional (non‐robust) standard errors. The outcome variable equals one if the person has completed university‐level education or is enrolled in university by the age of 26. Panel A displays the result for males and Panel B for low‐SES males. The basic models do not include any controls. The “Diet” model augments the basic model with diet score and quality of fat intake. The “Health” model augments the basic model with ideal cardiovascular health index and a variable indicating metabolic syndrome (MetS). The “Diet and health” model augments the basic model with both diet and health controls. The equality of intervention coefficients test used the intervention coefficient of the respective basic model as the baseline. Abbreviation: SES, socioeconomic status.

## 4 Discussion

Based on data from the randomized controlled STRIP study, we examined whether a 20‐year dietary intervention initiated in infancy was associated with university enrollment in adulthood and difficulties in school‐related tasks at age 10. Since many important milestones in child development occur in early childhood, early‐life interventions such as STRIP can have a significant impact on future educational achievement. Our results demonstrated that, on average, the associations between being in the intervention group and educational outcomes were small and statistically insignificant. However, for males, particularly those from low‐SES backgrounds, being in the intervention group was associated with fewer difficulties in school‐related tasks at the age of 10 and a 32% points higher probability of university enrollment, with a 95% CI of 0.103 to 0.546 indicating some uncertainty around this estimate. Compared to the introduction of free school lunches in Swedish primary schools, which increased the propensity to enter university by 1.5 percentage points in the overall population [15], the point estimate in our study is much higher. However, the confidence intervals are relatively wide, and the STRIP intervention was unusually intensive, involving individualized dietary counseling from infancy to age 20. Consequently, our estimates are not directly comparable to those from large‐scale population reforms, such as school meal programs. Moreover, our results are more likely to reflect the potential for substantial gains among particularly vulnerable groups, rather than an effect size that can be directly expected from scalable population‐wide programs. Our results aligned with a meta‐analysis that, while focusing on short‐term effects of school meal programs, found that the overall impact of such programs was generally small [14]. Consistent with the meta‐analysis, our findings also suggested that means‐tested schemes targeting students from low‐SES families may yield more favourable outcomes, and that females tend to show less pronounced benefits than males [14].

The importance of micronutrients for brain development and cognitive functioning is well documented [22, 23]. In addition to higher intakes of unsaturated relative to saturated fatty acids, participation in the STRIP intervention increased the intake of several key vitamins and minerals [18], suggesting that dietary improvements may have enhanced cognitive functioning and thereby mediated the relationship between the intervention and university enrollment. Consistent with this interpretation, participants in the STRIP intervention group demonstrated enhanced performance on cognitive tests assessing cognitive flexibility and inhibitory control [24]. These subdomains of executive functioning reflect the ability to manage conflicting information and ignore task‐irrelevant information. Enhanced executive functioning has been associated with improved academic performance [25, 26]. Moreover, a higher adherence to vegetables and dairy products has been linked to better cognitive function in the STRIP study [27]. Taken together, these findings suggest that higher cognitive functioning associated with participation in the STRIP intervention may have partly contributed to the observed increase in university enrollment among the participants. Cardiometabolic risk factors, such as elevated body mass index and high blood pressure, have also been linked to cognitive function and educational outcomes [28, 29, 30, 31, 32]. However, our findings suggest that the mediating role of cardiovascular health was comparatively less pronounced than that of diet. Another potential explanation is that the intervention as a whole, rather than any of its specific elements, altered children's attitudes towards university‐level education. For example, regular meetings with health care professionals may have increased children's interest in health care professions or higher education in general.

The higher university enrollment among low‐SES males exposed to the long‐term dietary intervention merits attention. Previous studies have documented differences in diet quality between males and females, as well as across socioeconomic statuses. Notably, females tend to pay greater attention to the health dimension of diet already early in life [33, 34], as also observed in the STRIP control group, where 9‐ to 11‐year‐old girls consumed more vegetables, fruits, and berries than boys, after adjusting for energy intake [35]. Also, lower socioeconomic status has been associated with poorer dietary outcomes [36, 37]. Consequently, males, and particularly those from lower‐SES backgrounds, may benefit more from improved diet in terms of cognitive functioning [22, 23], which could contribute to higher educational attainment.

Our study has limitations. First, our analysis was a *post hoc* extension of a randomized controlled trial originally designed to study cardiovascular outcomes, and the educational outcomes examined here were not primary endpoints of the intervention. Second, some dropout was unavoidable in a long‐term study like STRIP, which involved 45 visits for the intervention group and 28 for controls. Dropout was slightly higher in the intervention group, mainly due to moving, recurrent infections, or reluctance to provide blood samples. Comparisons between participants and dropouts have shown no differences in key health measures, components of metabolic syndrome, lifestyle factors, or parental socioeconomic status [38]. However, differences have been observed in dietary intake. Dropouts in the control group had higher energy, total fat, saturated fat, and cholesterol intake, as well as poorer fat quality than participants, whereas the opposite pattern was observed in the intervention group, suggesting that the beneficial effects of the intervention may be slightly underestimated [38]. Taken together, these observations, although based on a slightly different sample than that used in this study, suggest that non‐random attrition is quite unlikely to account for the observed effect. Nevertheless, its influence on the findings cannot be entirely ruled out, and it should be noted that no formal attrition‐adjustment methods were applied. In particular, some subgroup analyses were based on relatively small samples and, therefore, the estimates are best described as associations leveraging randomized assignment. Third, there are also potential limitations related to the mediation analyses in terms of timing of mediator measurement (measured between ages 15 and 17), possible residual confounding, and the reduced sample size for mediation analyses. Fourth, while the intervention was primarily diet‐focused, it also included additional guidance. Smoking prevention began at age 8, alongside counseling on maintaining a physically active lifestyle and avoiding alcohol and drug use. From age 10 onward, counseling expanded to self‐esteem, social relationships, identity, and decision‐making [39]. Although this type of counseling may influence educational outcomes, our findings suggested its impact was limited because children in the intervention group already exhibited fewer school difficulties by age 10, even though non‐dietary counseling began only at ages 8 and 10. Fifth, the study was conducted in Turku, one of Finland's university cities, and therefore may not be representative of the entire country. Nevertheless, the findings are generalizable to other urban regions in Finland. Sixth, the STRIP study involved personalized counseling over an extensive period, which poses a challenge for cost‐effective scaling of the intervention to large populations. However, because the STRIP intervention focused on counseling for heart‐healthy food choices rather than a strict diet, regular dietary guidance targeted at larger populations, such as in schools during early childhood, may yield in comparable outcomes. Seventh, although the core principles of the dietary guidelines have remained stable, minor changes over time may have influenced particular nutrient intakes and should be considered when interpreting the results. It should also be noted that the effects observed in this study may differ under current guidelines. Nevertheless, our findings suggest that dietary counseling aimed at replacing saturated fats with unsaturated fats, increasing the consumption of whole grains, fruits, and vegetables, and reducing sodium and added sugar intake may support educational attainment. Finally, with respect to measurement validity, key behavioral variables, including smoking, leisure‐time physical activity, and dietary intake, were self‐reported by adolescents and may be subject to reporting bias. In contrast, biomarkers were measured using standardized procedures by trained healthcare professionals, minimizing the risk of measurement error.

The main strength of this study lies in its RCT setting, which serves to mitigate bias. Moreover, the STRIP study enabled us to explore the association between a dietary intervention initiated in infancy and long‐term educational outcomes. We also examined the extent to which diet and health markers might explain the connection between the intervention and university enrollment.

## 5 Conclusions

Beyond the STRIP intervention, our findings suggest broader implications for public health and education policy. Early, family‐based dietary counseling may not only improve health but also support cognitive development linked to later educational success. Education is a fundamental determinant of future socioeconomic status and health outcomes [40, 41]. Integrating preventive health programs into child and youth policy could therefore yield long‐term benefits that extend beyond health, enhancing educational and societal outcomes. Such policies may also promote equality by increasing university enrollment among populations where higher education levels are less prevalent, such as males and individuals with lower family SES in Finland. Previous studies have shown that implementing healthy school meals has a positive impact on children's long‐term educational outcomes [15]. In Finland, all pupils and students attending pre‐primary, basic, and upper secondary education are entitled to a full meal free of charge. Our findings suggested that providing long‐term dietary counseling to children and families, even with ample nutrition at school, can further enhance children's educational attainment and future outcomes in a high‐income country.

## Author Contributions

**Jutta Viinikainen:** conceptualization, writing – original draft, methodology, writing – review and editing, formal analysis. **Guido Heineck:** conceptualization, methodology, writing – review and editing. **Petri Böckerman:** conceptualization, methodology, writing – review and editing. **Katja Pahkala:** conceptualization, writing – review and editing. **Antti Jula:** conceptualization, writing – review and editing. **Jaana T. Kari:** conceptualization, writing – review and editing. **Hanna Lagström:** conceptualization, writing – review and editing. **Harri Niinikoski:** conceptualization, writing – review and editing. **Jaakko Pehkonen:** conceptualization, writing – review and editing. **Suvi P. Rovio:** conceptualization, writing – review and editing. **Tapani Rönnemaa:** conceptualization, writing – review and editing. **Pia Salo:** conceptualization, writing – review and editing. **Jorma Viikari:** conceptualization, writing – review and editing. **Olli Raitakari:** conceptualization, writing – review and editing, project administration, resources.

## Ethics Statement

This study involved human participants. Written informed consent was obtained from the parents at the beginning of the study and from the children at ages 15 and 18. The study follows the Declaration of Helsinki and was approved by the Joint Commission on Ethics of Turku University and Turku University Central, and the trial is registered with ClinicalTrials.gov (#NCT00223600). All authors have read and approved the final version of the manuscript. Jutta Viinikainen had full access to all of the data in this study and takes complete responsibility for the integrity of the data and the accuracy of the data analysis. This manuscript has been presented at the following venues: CHE Seminar Series (2019), Summer Seminar for Finnish Economists (2019), Annual Meeting of the Finnish Economic Association (2020), 42nd Nordic Health Economics Study Groups meeting (2023).

## Conflicts of Interest

The authors declare no conflicts of interest.

## Transparency Statement

The lead author Jutta Viinikainen affirms that this manuscript is an honest, accurate, and transparent account of the study being reported; that no important aspects of the study have been omitted; and that any discrepancies from the study as planned (and, if relevant, registered) have been explained.

## Supporting information


**Supporting File:** hsr272359‐sup‐0001‐Supporting_information.docx.

## Data Availability

The data that support the findings of this study are not publicly available due to privacy or ethical restrictions. Data sharing outside the STRIP group requires a data‐sharing agreement and approval from the STRIP Steering Committee. Interested researchers may submit an expression of interest to the STRIP Steering Committee for consideration. Stata codes used in the analyses are available upon request from the corresponding author.
