# Supplementary material for: Participation in a 20‐Year Randomized Dietary Trial and University Enrollment
Source: Health Sci Rep. 2026 Apr 19;9(4):e72359. doi: 10.1002/hsr2.72359 (PMC13092225; doi:10.1002/hsr2.72359)
Supplement: Supplementary file 1 — Supporting File: hsr272359‐sup‐0001‐Supporting_information.docx. [file HSR2-9-e72359-s001.docx]

Supporting information

Table S1. Recommended dietary fat intake in the Nordic nutrition recommendations (1996–2023).

| Year | Total fat | SAFA | MUFA | PUFA | n-6/n-3 |
| --- | --- | --- | --- | --- | --- |
| 1996 | 30 to 35 E% | <10E% | 10–15E% | 10E% | Recommendations for fat type are the same as for adults, except that n-6 fatty acids should provide at least 4.5 E% for children ≤1 year and ≥3 E% for ages 1–3 years. The minimum n-3 fatty acid intake should be 0.5 E% for children <3 years. |
| 2004 | 25–35 E% | <10E% | 10–15 E% | 5–10E% | n-6 + n-3 ≥3 E%, including ≥0.5 E% from n-3. |
| 2012 | 25–40E% | <10E% | 10–20E% | 5–10E% | Linoleic (n-6) and alpha-linolenic (n-3) acids are essential and should contribute at least 3 E%, including at least 0.5 E% as alpha-linolenic acid. |
| 2023 | 25–40E% | <10E% | 10–20E% | 5–10E% | Of PUFA, n-6/n-3/ALA should be at least 3/1/0.5 E% |

^Abbreviations: SAFA – saturated fatty acids; MUFA – monounsaturated fatty acids; PUFA – polyunsaturated fatty acids; n-6 – linoleic acid; n-3 – omega-3 fatty acids; ALA – alpha-linolenic acid; E% – percentage of daily energy intake.^

Table S2. Dietary intervention participation and university enrollment. Results from a logit model.

|  | Average marginal effects | P-value | 95% CI |
| --- | --- | --- | --- |
| **Panel A**: All (n = 639) |  |  |  |
| Intervention | 0.031 | 0.427 | -0.046, 0.108 |
| **Panel B**: All (n = 639) |  |  |  |
| Intervention | 0.105 | 0.065 | -0.006, 0.216 |
| Intervention × female | -0.138 | 0.074 | -0.290, 0.013 |
| **Panel C**: Low SES (n = 153) |  |  |  |
| Intervention | 0.313 | 0.003 | 0.103, 0.523 |
| Intervention × female | -0.489 | 0.000 | -0.762, -0.215 |
| **Panel D**: High SES (n = 354) |  |  |  |
| Intervention | 0.032 | 0.674 | -0.119, 0.184 |
| Intervention × female | -0.084 | 0.421 | -0.288, 0.120 |

^The table reports average marginal effects, P-values, and 95% confidence intervals using heteroskedasticity-robust standard errors from a regression of university enrollment on random intervention assignment (1 for individuals in the intervention group and 0 otherwise). The outcome variable equals one if the person has completed university-level education or is enrolled in university by the age of 26. Panels B-D include a sex indicator (male = 0, female = 1) and an interaction term (Intervention × female) as additional controls.^ ^Abbreviations: SES, socioeconomic status. Families with at least one parent who had completed university-level education by the time the child was five years old are considered high SES families, while those without such a parent are considered low SES families.^
